# Supplementary material for: The GPR171 pathway suppresses T cell activation and limits antitumor immunity
Source: Nat Commun. 2021 Oct 6;12:5857. doi: 10.1038/s41467-021-26135-9 (PMC8494883; doi:10.1038/s41467-021-26135-9)
Supplement: Supplementary file 1 — Supplementary Information [file 41467_2021_26135_MOESM1_ESM.pdf]

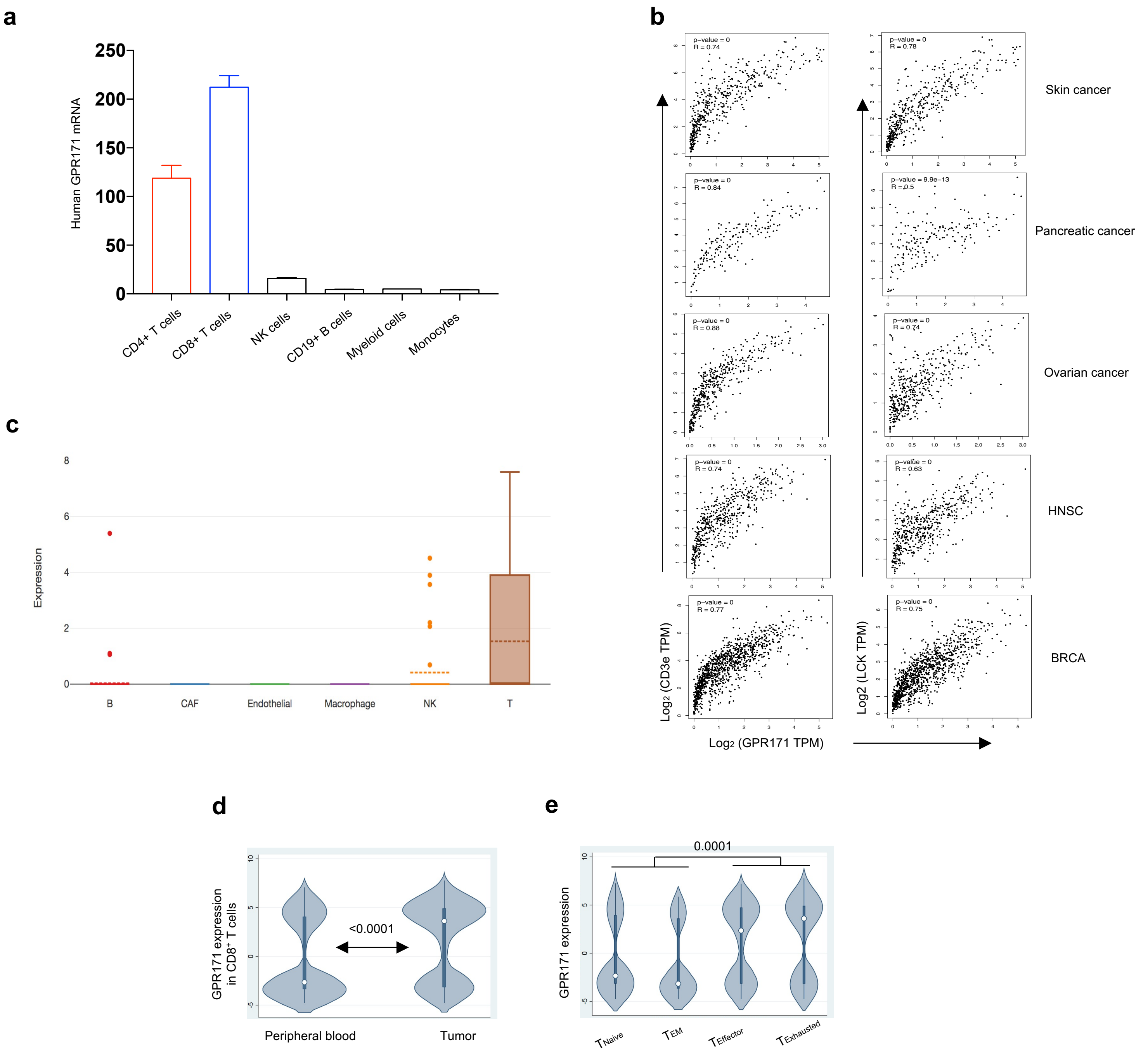

Supplementary Figure 1 ***GPR171 is preferentially expressed in human T cells.***

(a) The BioGPS microarray database indicated that GPR171 is primarily expressed in T cells. (b) In the TCGA dataset, the expression of GPR171 is well correlated with other T cell signature genes, including *CD3e* and *LCK*. HNSC, head and neck squamous cell carcinoma; BRCA, breast invasive carcinoma. (c) In a single-cell RNAseq dataset of immune cells within human melanoma tissue, GPR171 transcript was enriched in NK and T cells. Box-and-whisker showed the median (dotted line), 25th and 75th percentiles (box) and 5th and 95th percentiles (whiskers). (d, e) GPR171 expression levels in CD8+ T cells from peripheral blood or tumor (d), as well as in different phenotypes of CD8+ T cell in liver tumor (e). Violin plots represented the density distribution and the boxplots within the median (circle), 25th and 75th percentiles (box) and 5th and 95th percentiles (whiskers).

Statistical significance was determined by two-tailed Student's t-test for d and e. Unless otherwise denoted, values are mean ± SEM.

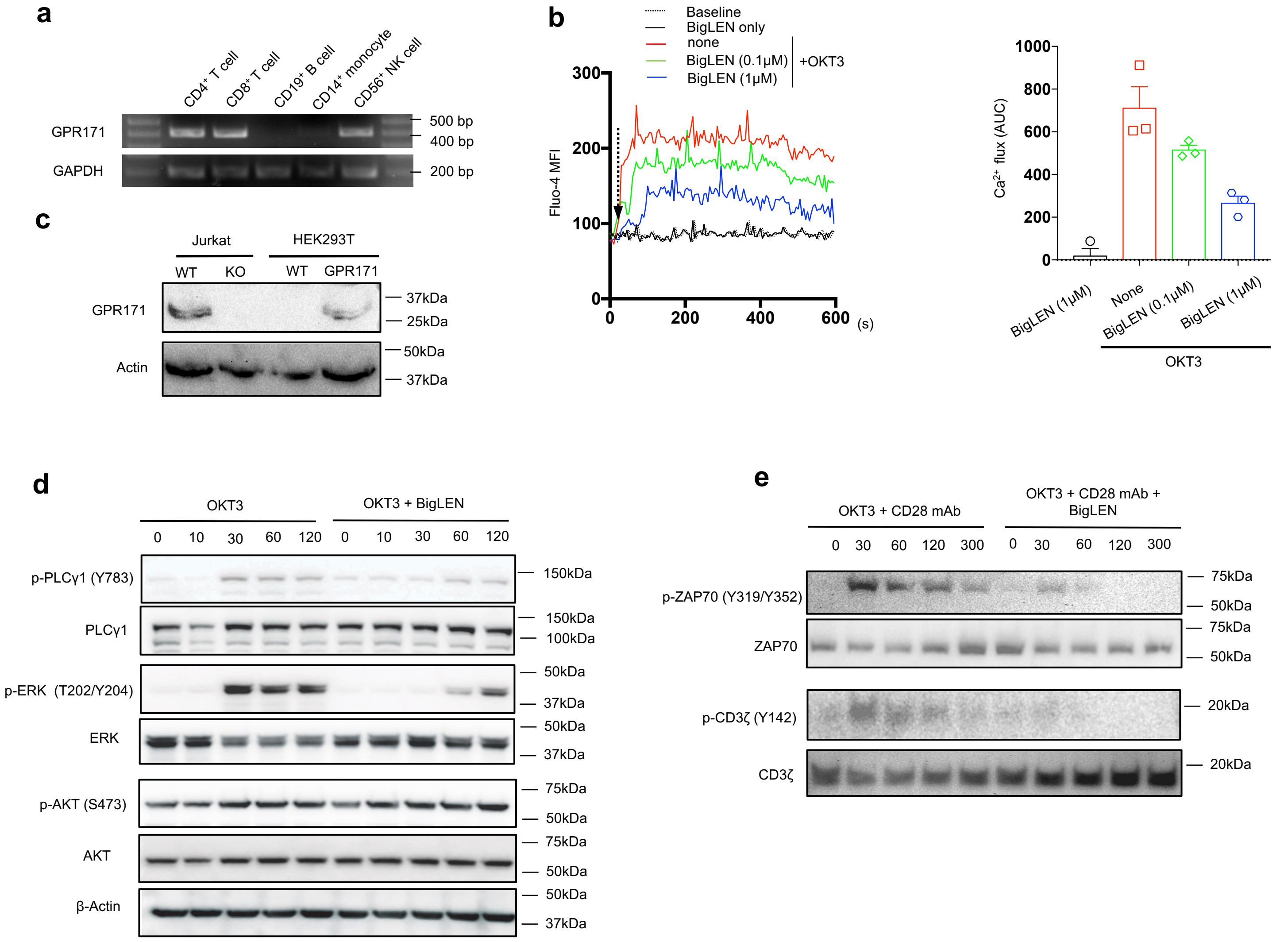

Supplementary Figure 2 ***GPR171 signaling inhibits T cell response.***

(a) GPR171 transcript in different immune cell types isolated from peripheral blood of healthy donor by RT-PCR. (b) Intracellular calcium concentration of PBMC was measured upon the treatment of BigLEN, biotin-human CD3 mAb (5μg/ml) and streptavidin crosslinking (20μg/ml) (Left). The calcium flux was measured by area under the curve (AUC) (Right). (c) GPR171 protein in WT or $GPR171^{KO}$ Jurkat cells was detected by western blot. (d) Phosphorylated PLCγ1, ERK and AKT in human PBMC cocultured with BigLEN (1μM) and stimulated with human CD3 mAb (OKT3, 0.5μg/ml) were determined by western blot. (e) Phosphorylated ZAP70 and CD3ζ in human PBMC cocultured with BigLEN (1μM) and stimulated with human CD3 mAb (OKT3, 1μg/ml) plus human CD28 mAb (0.01μg/ml) were determined by western blot.

Unless otherwise denoted, values are mean ± SEM. Source data was provided as a Source Data file. All data are representative of two independent experiments.

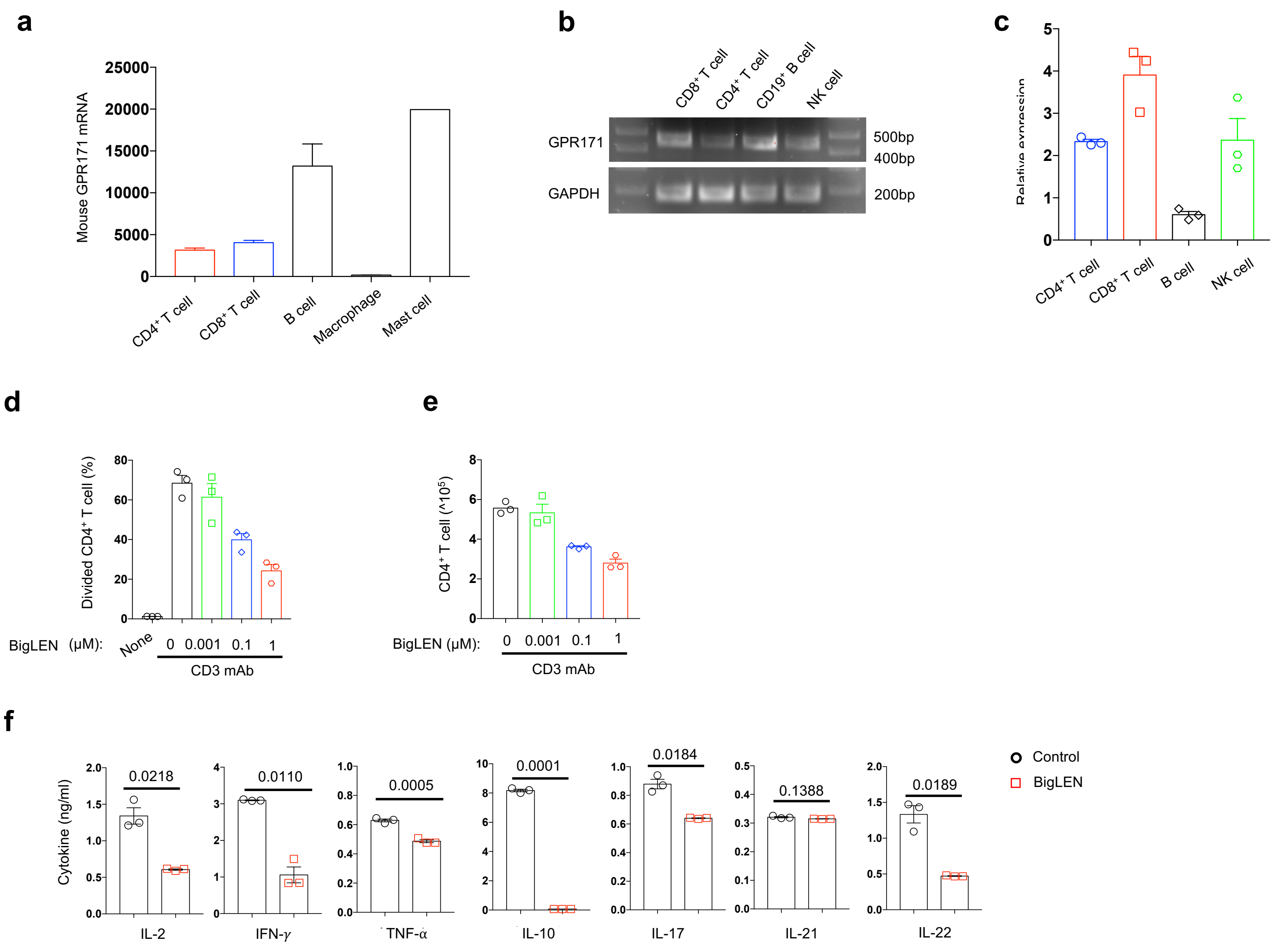

Supplementary Figure 3 ***GPR171 signaling inhibits mouse T cell response in vitro.***

(a) The BioGPS microarray database indicated that mouse GPR171 is expressed in T cells, B cells, and mast cells. Data are mean ± SEM. (b, c) GPR171 expression in different immune cells sorted from naïve mouse spleen was determined by RT-PCR (b) and quantified by qPCR (c). (d-f) CFSE-labeled CD4+ T cells were stimulated by plate- coated mouse CD3 mAb with or without BigLEN for 6 days. The percentage of divided T cells (d) and the numbers of live T cells (e) were determined. Cytokines in the supernatant was quantified (f). Except IL-2 (1 day), all other cytokines were quantified in the supernatant 6 days post- culture. (f) n=3 biologically independent samples.

Statistical significance was determined by two-tailed Student's t-test for f. Unless otherwise denoted, values are mean ± SEM. Source data was provided as a Source Data file. Data (b-f) are representative of two independent experiments.

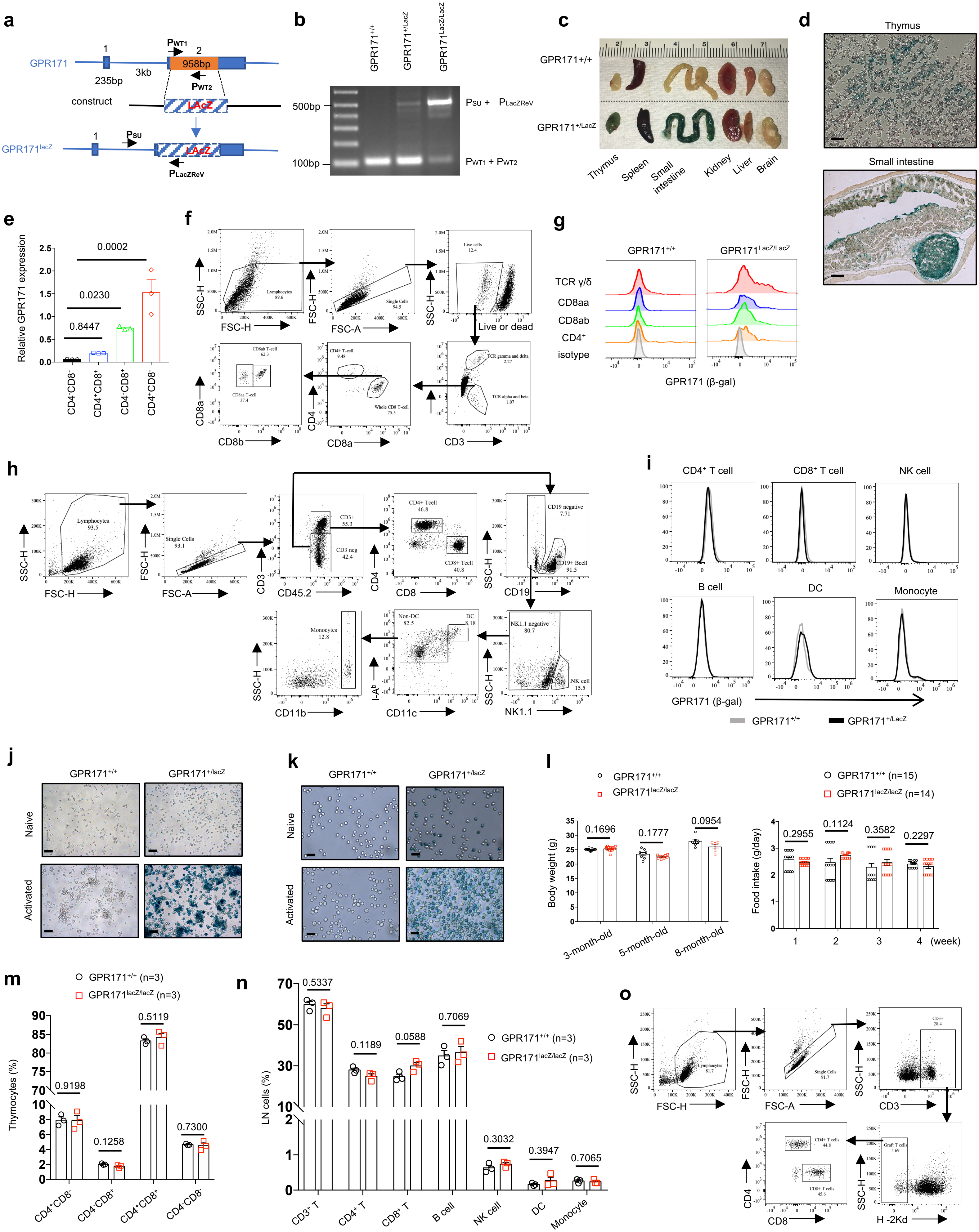
a
GPR171
1
235bp
3kb
PWT1
2
958bp
PWT2
construct
LAcZ
GPR171lacZ
PSU
PLacZReV
b
GPR171+/+
GPR171+/LacZ
GPR171LacZ/LacZ
500bp
100bp
PSU + PLacZReV
PWT1 + PWT2
c
GPR171+/+
GPR171+/LacZ
Thymus
Spleen
Small intestine
Kidney
Liver
Brain
d
Thymus
Small intestine
e
Relative GPR171 expression
0.0002
0.0230
0.8447
CD4-CD8-
CD4+CD8+
CD4-CD8+
CD4+CD8-
f
SSC-H
FSC-H
FSC-A
Lymphocytes
Single Cells
Live cells
Live or dead
TCR gamma and delta
TCR alpha and beta
CD8ab T-cell
CD8aa T-cell
CD4+ T-cell
Whole CD8 T-cell
CD8a
CD8b
CD4
CD3
g
GPR171+/+
GPR171LacZ/LacZ
TCR γ/δ
CD8aa
CD8ab
CD4+
isotype
GPR171 (β-gal)
h
Lymphocytes
Single Cells
CD3+
CD3 neg
CD4+ Tcell
CD8+ Tcell
CD19 negative
CD19+ Bcell
NK1.1 negative
NK cell
Non-DC
DC
Monocytes
SSC-H
FSC-H
FSC-A
CD3
CD45.2
CD4
CD8
CD19
CD11b
I-Ab
CD11c
NK1.1
i
CD4+ T cell
CD8+ T cell
NK cell
B cell
DC
Monocyte
GPR171 (β-gal)
GPR171+/+
GPR171+/LacZ
j
GPR171+/+
GPR171+/lacZ
Naive
Activated
k
GPR171+/+
GPR171+/lacZ
Naive
Activated
l
GPR171+/+
GPR171lacZ/lacZ
Body weight (g)
0.1696
0.1777
0.0954
3-month-old
5-month-old
8-month-old
GPR171+/+ (n=15)
GPR171lacZ/lacZ (n=14)
Food intake (g/day)
0.2955
0.1124
0.3582
0.2297
1
2
3
4
(week)
m
GPR171+/+ (n=3)
GPR171lacZ/lacZ (n=3)
Thymocytes (%)
0.9198
0.1258
0.5119
0.7300
CD4+CD8-
CD4-CD8+
CD4+CD8+
CD4-CD8-
n
LN cells (%)
0.5337
0.1189
0.0588
0.7069
0.3032
0.3947
0.7065
GPR171+/+ (n=3)
GPR171lacZ/lacZ (n=3)
CD3+ T
CD4+ T
CD8+ T
B cell
NK cell
DC
Monocyte
o
Lymphocytes
Single Cells
CD3+
CD4+ T cells
CD8+ T cells
Graft T cells
SSC-H
FSC-H
FSC-A
CD3
CD4
CD8
H -2Kd

Supplementary Figure 4 ***Generating and characterizing GPR171$^{LacZ/LacZ}$ mice.***

(a) The strategy of generating GPR171$^{lacZ/lacZ}$ mice. The protein coding sequences of mouse GPR171 was replaced by a *lacZ* reporter. (b) Genomic PCR was performed to screen GPR171$^{+/+}$ (WT), GPR171$^{+/lacZ}$, or GPR171$^{lacZ/lacZ}$ mice. The genomic positions for PCR Primers are indicated in A. (c) X-gal staining was performed to detect GPR171 expression in different organs of WT and GPR171$^{+/lacZ}$ mice. (d) Representatives of X-gal staining of thymus and small intestine tissues from GPR171$^{+/lacZ}$ mice. (e) GPR171 transcript in different thymocytes from naïve B6 mice was determined by qPCR. (f) Gate strategy to sort TCRγ/δ positive or CD8ab T cells in intestine. (g) GPR171 (β-gal) expression in splenic immune cells from naïve WT or GPR171$^{+/lacZ}$ mice by flow cytometry. (h) Gate strategy to sort various types of immune cells in spleen. (i) GPR171 (β-gal) expression in different T cell subsets of small intestine from naïve WT or GPR171$^{lacZ/lacZ}$ mice by flow cytometry. (j) Naïve or activated splenocytes by coated CD3 and CD28 mAbs from WT or GPR171$^{+/lacZ}$ mice were stained for X-gal. (k) Purified naïve NK cells or activated NK cells from WT or GPR171$^{+/lacZ}$ mice were stained for X-gal. Activated splenic NK cells were isolated from mice injected with poly I:C overnight. (l-n) Normal body weight and T cell development in GPR171$^{lacZ/lacZ}$ mice. Body weight (left, 3-month-old; WT: n=15, GPR171$^{lacZ/lacZ}$: n=14, 5-month-old; WT: n=8, GPR171$^{lacZ/lacZ}$: n=9, 8-month-old; WT: n=6, GPR171$^{lacZ/lacZ}$: n=6) and food intake (right, WT: n=15, GPR171$^{lacZ/lacZ}$: n=14) were similar between WT and GPR171$^{lacZ/lacZ}$ mice (l). Immune cell composition in thymus (m) and lymph nodes (n) from 8-week-old GPR171$^{lacZ/lacZ}$ mice and WT littermates were determined by flow cytometry. No significant difference was found between WT and GPR171$^{lacZ/lacZ}$ mice by two-tailed Student's t-test. (e, m, n) n=3 biologically independent samples. (o) Gate strategy to analyze grafted T cells (H-2K^b) from peripheral blood and spleen in the GVH model. (d, j, k) Scale bar, 20μm.

Statistical significance was determined by One-way ANOVA for e and two-tailed Student's t-test for l, m and n. . Unless otherwise denoted, values are mean ± SEM. Source data was provided as a Source Data file. Data are representative of two (e, g, i, j, k, l, m and n) or three independent experiments (b, c, and d).

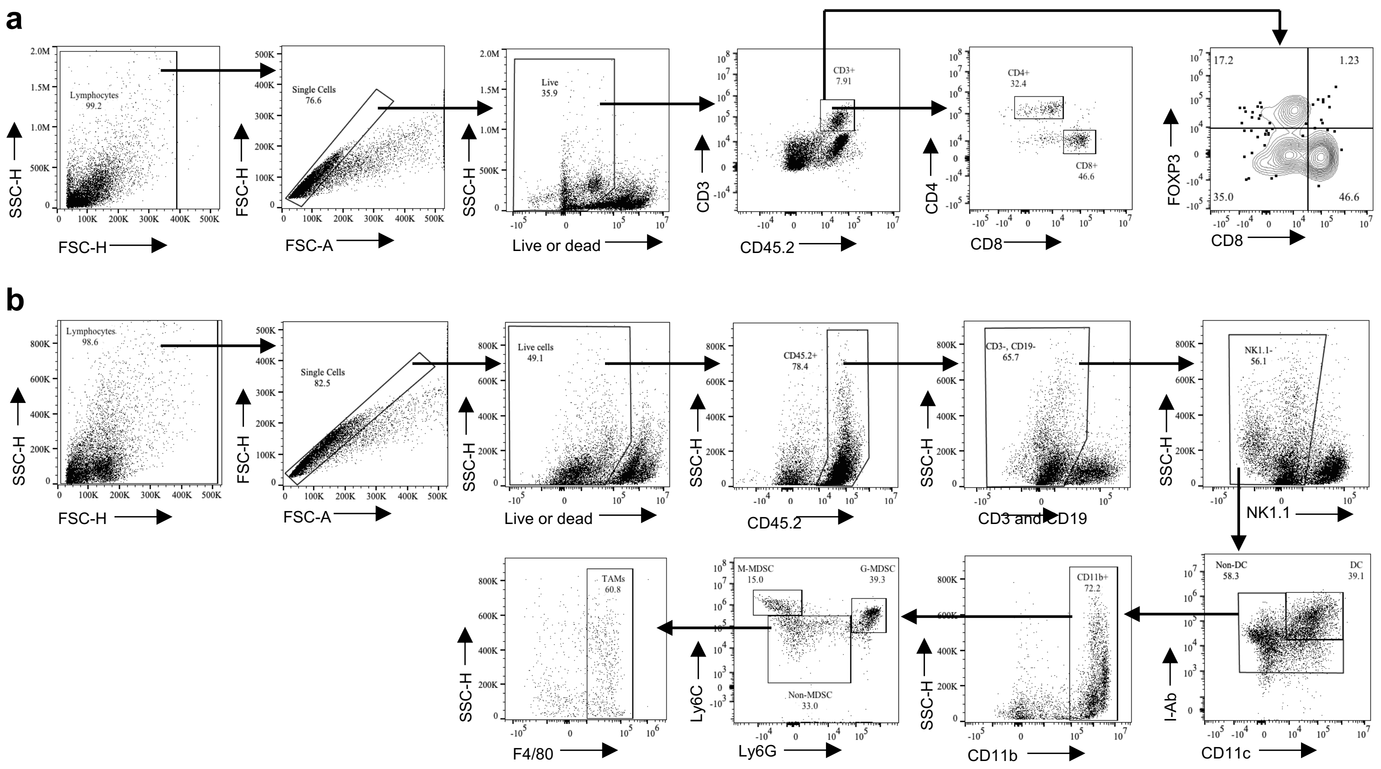

Supplementary Figure 5 Gating strategies

Gating strategy to sort CD8^{+} or CD4 T^{+} cells and Treg (CD45.2^{+}, CD3^{+}, CD8^{-}, CD4^{+}, FOXP3^{+}) in MC38 tumor (a). Gate strategy to NK cells (CD45.2^{+}, CD3^{-}, CD19^{-}, NK1.1^{+}), Dendritic cells (CD45.2^{+}, CD3^{-}, CD19^{-}, NK1.1^{-}, I-Ab+, CD11c^{+}), Granulocytic-MDSC (CD45.2^{+}, CD3^{-}, CD19^{-}, NK1.1^{-}, CD11b^{+}, Ly-6G^{+}, Ly-6C^{+}), Monocytic-MDSC (CD45.2^{+}, CD3^{-}, CD19^{-}, NK1.1^{-}, CD11b^{+}, Ly-6G^{-}, Ly-6C^{+}), and TAM (CD45.2^{+}, CD3^{-}, CD19^{-}, NK1.1^{-}, CD11b^{+}, Ly-6G^{-}, Ly-6C^{-}, F4/80^{+}), in MC38 tumor (b).

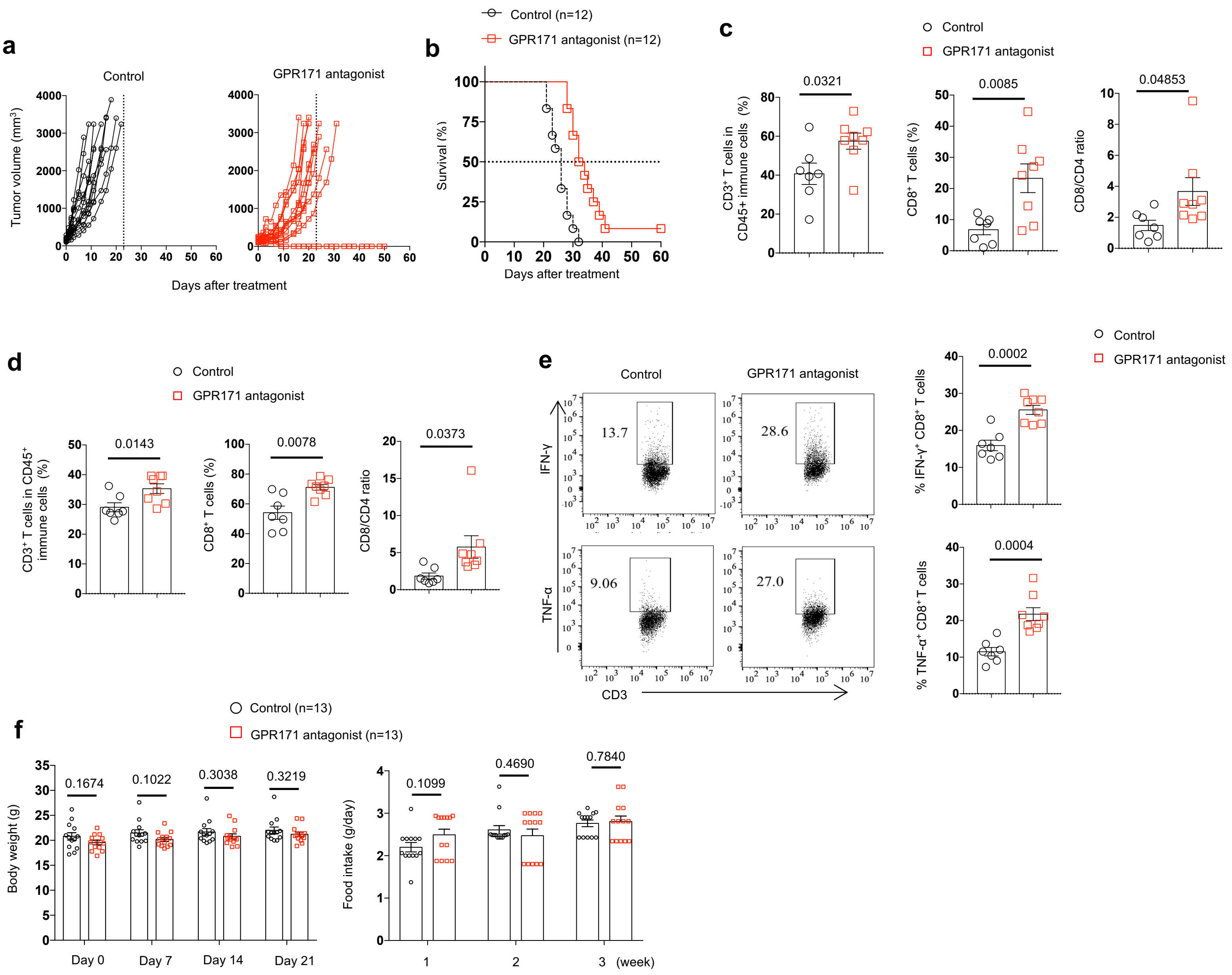

Supplementary Figure 6 ***GPR171 blockade promotes antitumor T cell functions in mouse tumor models.***

(a, b) 10 days after MC38 tumor inoculation, B6 mice were started with the treatment of GPR171 antagonist or control three times per week for total 9 times. Tumor growth (a) and mouse survival (b) were followed up to 60 days post-treatment (n=12, each). (c) Immune cell composition of B16-OVA under the treatment of GPR171 antagonist. Single-cell suspensions were prepared from tumors 14 days after tumor inoculation. The percentages of intratumoral CD3+ T cells, CD8+ T cells, as well as the ratio of CD8+ T to CD4+ T cells were determined by flow cytometry (Control: n=7, GPR171 antagonist: n=8). (d) In CT26 tumor model under the treatment of GPR171 antagonist, single-cell suspensions were prepared from tumors 21 days after tumor inoculation. The percentages of intratumoral CD3+ T cells, CD8+ T cells, as well as the ratio of CD8+ T to CD4+ T cells were determined by flow cytometry. (e) The percentage of IFN-γ and TNF-α -producing cells in CD8+ TILs in CT26 tumor stimulated by AH1 peptide. (Control: n=7, GPR171 antagonist: n=8). (f) Body weight and food intake by GPR171 antagonist treatment in tumor-bearing mice (n=13, each).

Statistical significance was determined by two-tailed Student's t-test for c, d and e, or Log-rank test for b. Unless otherwise denoted, values are mean ± SEM. Source data was provided as a Source Data file. Data are representative of three (a and b) or two independent experiments (c, d, e and f).

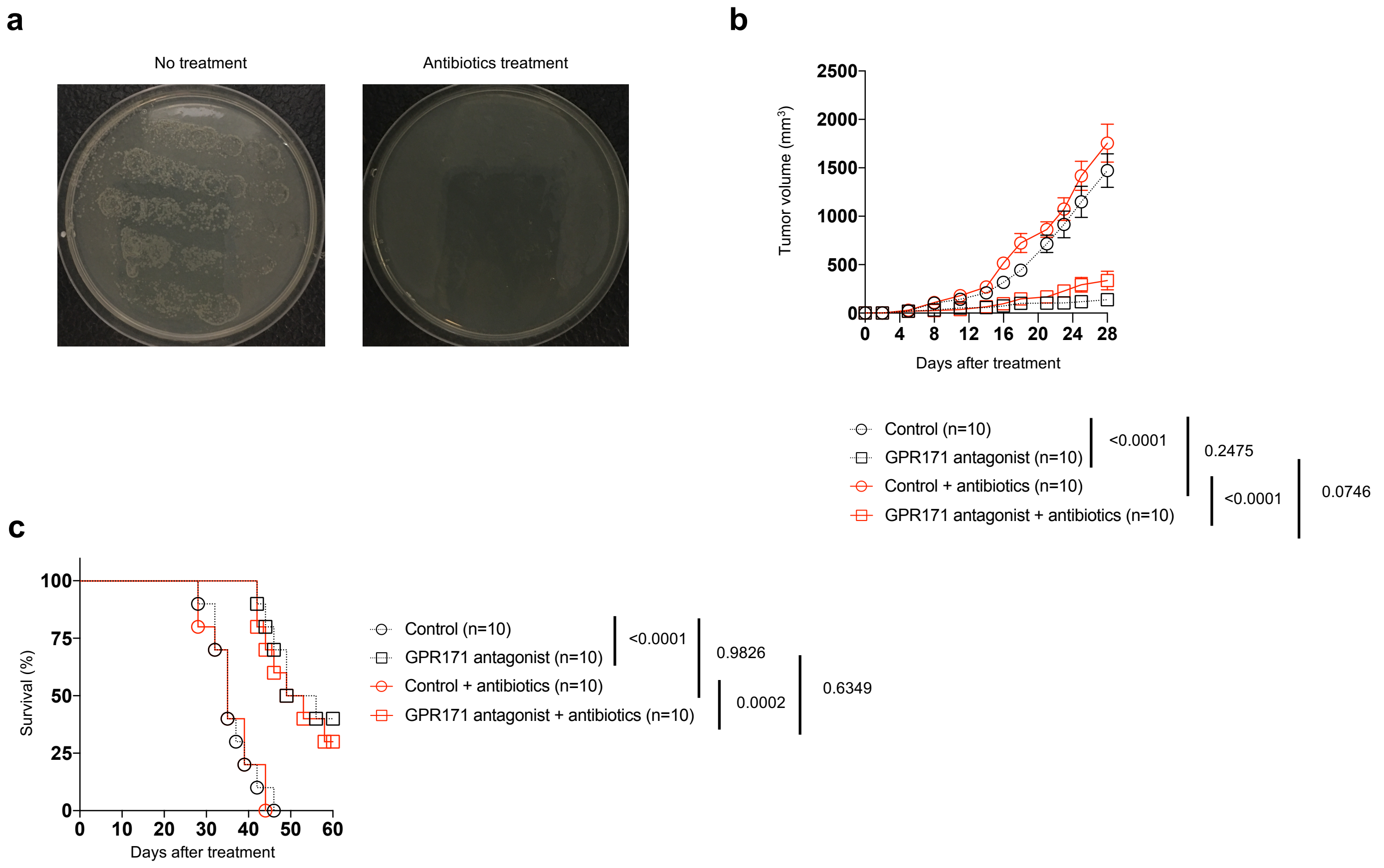

Supplementary Figure 7 ***The involvement of microbiota in GPR171 antagonist-triggered antitumor immunity.***

(a) Fecal cultures were performed after mice were under antibiotics treatment for two weeks. After antibiotics treatment, B6 mice were inoculated MC38 and then started with the treatment of GPR171 antagonist or control three times per week. Tumor growth (b) and mouse survival (c) were followed (n=10, each).

Statistical significance was determined by Two-way ANOVA for b and Log-rank test for c. Unless otherwise denoted, values are mean ± SEM. Source data was provided as a Source Data file. Data are representative of two independent experiments (a, b and c).

	Name	Company	Clone	Catalog #
**1**	Anti-mouse CD8β mAb	BioXcell	53-5.8	BE0223
**2**	Anti-mouse CD4 mAb	BioXcell	GK1.5	BE0003-1
**3**	Anti-mouse CTLA-4 mAb	BioXcell	9D9	BE0164
**4**	Anti-mouse TIGIT mAb	BioXcell	1G9	BE0274

Supplementary table 1. Information of antibodies for vivo experiments

	Gene name		Sequence (5'-3')
**RT-PCR or qPCR**	Human GPR171	Sense	GGAGCGAGATCCCTCCAAAAT
		Antisense	GGCTGTTGTCATACTTCTCATGG
	Mouse GPR171	Sense	AGGCCAGAGACGACACAGCCA
		Antisense	CCAGGGTGCCAGACCCAAGT
	Human GAPDH	Sense	GGAGCGAGATCCCTCCAAAAT
		Antisense	GGCTGTTGTCATACTTCTCATGG
	Mouse GAPDH	Sense	TGGCCTTCCGTGTTCCTA
		Antisense	GAGTTGCTGTTGAAGTCGCA
**Genotyping**	GPR171 WT	P_{WT1}	TGGCACCCTGGAAGCTAAG
		P_{WT2}	CGATGCTGACAAAGGCTAAGAAG
	GPR171 mutant	P_{SU}	CGCAAAGAAATGTCTCCCTC
		$P_{LacZRev}$	GTCTGTCCTAGCTTCCTCACTG

Supplementary table 2. Primer-lists

	Name	Company	Clone	Catalog #
**1**	Phosphorylated PLCγ-1 (Tyr783)	Cell signaling		2821
**2**	PLCγ1	Cell signaling	D9H10	8713S
**3**	Phosphorylated p44/42 (ERK1/2) (Thr202/Tyr204)	Cell signaling	D13.14.4E	4370S
**4**	p44/42 MAPK	Cell signaling	137F5	4965S
**5**	Phosphorylated AKT (Ser473)	Cell signaling	D9E	4060S
**6**	AKT	Cell signaling	C67E7	4691S
**7**	Phosphorylated ZAP70 (Tyr319/Tyr352)	Cell signaling	65E 4	2717S
**8**	ZAP70	Cell signaling	99F2	2705S
**9**	Phosphorylated CD3ζ (Tyr142)	Cell signaling	Y142	67748S
**10**	β-actin	Cell signaling	8H10D10	3700S
**11**	CD3ζ	Santa Cruz	6B10.2	sc-1239
**12**	Anti-human CD3 mAb	Biolegend	OKT3	317326
**13**	Anti-mouse CD3 mAb	Biolegend	145-2C11	100340
**14**	Anti-mouse CD28 mAb	eBioscience	CD28.6	16-0288-81

Supplementary table 3. Information of antibodies for Western blot

		Name	Company	Clone	Catalog #
**1**	Mouse	CD3ε	Biolegend	145-2C11	100308
**2**	Mouse	CD3	Biolegend	17A2	100236
**3**	Mouse	CD11b	Biolegend	M1/70	101208/101212
**4**	Mouse	CD69	Biolegend	H1.2F3	104507
**5**	Mouse	PD-1	Biolegend	RMP1-30	109104
**6**	Mouse	TIGIT	Biolegend	1G9	142103
**7**	Mouse	LAG3	Biolegend	C9B7W	125207
**8**	Mouse	TIM3	Biolegend	RMT3-23	119703
**9**	Mouse	IFNγ	Biolegend	XMG1.2	505807
**10**	Mouse	CD137 (41-BB)	Biolegend	17B5	106106
**11**	Mouse	FOXP3	Biolegend	150D	320008
**12**	Mouse	TCRγ/δ	Biolegend	GL3	118107
**13**	Mouse	H-2Kb	Biolegend	AF-688.5	116517
**14**	Mouse	H-2Kd	Biolegend	SF1-1.1	116623
**15**	Mouse	TNFα	Biolegend	MP6-XT22	506307
**16**	Mouse	CD8a	Biolegend	53-6.7	100712/100738/100748
**17**	Mouse	CD11c	Biolegend	N418	117343
**18**	Mouse	CD4	Biolegend	RM4-5	100510/100559
**19**	Mouse	CD45.2	Biolegend	104	109841
**20**	Mouse	NK1.1	Biolegend	PK136	108707/108713/108737
**21**	Mouse	I-Ab	Biolegend	AF6-120.1	116419
**22**	Mouse	Ly-6G	Biolegend	1A8	127606
**23**	Mouse	Ly-6C	Biolegend	HK1.4	128011
**24**	Mouse	F4/80	Biolegend	BM8	123147
**25**	Mouse	CD45.1	Biolegend	A20	110706/110714
**26**	Mouse	CD8b	Biolegend	YTS156.7.7	126605
**27**	Mouse	CD16/32	Biolegend	93	101330
**28**	Mouse	CD19	Biolegend	6D5	115512/115545/115555
**29**	Mouse	Granzyme B	Biolegend	QA18A28	396404
**30**		CFSE	Biolegend		422701
**31**	Human	CD8	Biolegend	HIT8a	300908/300912
**32**	Human	CD4	Biolegend	OKT4	317410/317416
**33**	Human	CD3	Biolegend	HIT3a	300308
**34**	Human	CD45	Biolegend	HI30	304026
**35**		β-galactosidase	Cell signaling	8H10D10	3700S

Supplementary table 4. Information of antibodies for Flowcytometry
