# Supplementary material for: The GPR171 pathway suppresses T cell activation and limits antitumor immunity
Source: Nat Commun. 2021 Oct 6;12:5857. doi: 10.1038/s41467-021-26135-9 (PMC8494883; doi:10.1038/s41467-021-26135-9)
Supplement: Supplementary file 3 — Reporting Summary [file 41467_2021_26135_MOESM3_ESM.pdf]

nature research

Corresponding author(s): Yuwen Zhu

Last updated by author(s): June/30/21

Reporting Summary

Nature Research wishes to improve the reproducibility of the work that we publish. This form provides structure for consistency and transparency in reporting. For further information on Nature Research policies, see our Editorial Policies and the Editorial Policy Checklist.

Statistics

For all statistical analyses, confirm that the following items are present in the figure legend, table legend, main text, or Methods section.

n/a	Confirmed	
☐	☒	The exact sample size (*n*) for each experimental group/condition, given as a discrete number and unit of measurement
☐	☒	A statement on whether measurements were taken from distinct samples or whether the same sample was measured repeatedly
☐	☒	The statistical test(s) used AND whether they are one- or two-sided<br>*Only common tests should be described solely by name; describe more complex techniques in the Methods section.*
☐	☒	A description of all covariates tested
☐	☒	A description of any assumptions or corrections, such as tests of normality and adjustment for multiple comparisons
☐	☒	A full description of the statistical parameters including central tendency (e.g. means) or other basic estimates (e.g. regression coefficient) AND variation (e.g. standard deviation) or associated estimates of uncertainty (e.g. confidence intervals)
☐	☒	For null hypothesis testing, the test statistic (e.g. *F*, *t*, *r*) with confidence intervals, effect sizes, degrees of freedom and *P* value noted<br>*Give P values as exact values whenever suitable.*
☒	☐	For Bayesian analysis, information on the choice of priors and Markov chain Monte Carlo settings
☒	☐	For hierarchical and complex designs, identification of the appropriate level for tests and full reporting of outcomes
☒	☐	Estimates of effect sizes (e.g. Cohen's *d*, Pearson's *r*), indicating how they were calculated

Our web collection on statistics for biologists contains articles on many of the points above.

Software and code

Policy information about availability of computer code

Data collection	FlowJo software
Data analysis	IBM SPSS statistics 24 version software for Mac and GraphPad Prism 7.0 software (GraphPad Software).

For manuscripts utilizing custom algorithms or software that are central to the research but not yet described in published literature, software must be made available to editors and reviewers. We strongly encourage code deposition in a community repository (e.g. GitHub). See the Nature Research guidelines for submitting code & software for further information.

Data

Policy information about availability of data

All manuscripts must include a data availability statement. This statement should provide the following information, where applicable:

- Accession codes, unique identifiers, or web links for publicly available datasets
- A list of figures that have associated raw data
- A description of any restrictions on data availability

We have included the information about the sources of data-sets which we got for analysis in our manuscript.
Suppel Figure 1A: The BioGPS microarray database(http://biogps.org/#goto=welcome).
Suppel Figure 1B: The Cancer Genome Atlas (TCGA) database (https://www.cancer.gov/about-nci/organization/ccg/research/structural-genomics/tcga)
Suppel Figure 1C: Tirosh, I., Izar, B., Prakadan, S.M., Wadsworth, M.H., 2nd, Treacy, D., Trombetta, J.J., Rotem, A., Rodman, C., Lian, C., Murphy, G., et al. (2016). Dissecting the multicellular ecosystem of metastatic melanoma by single-cell RNA-seq. Science 352, 189-196.
Suppel Figure 1D: Zhang, Q., et al. (2017). Landscape of Infiltrating T Cells in Liver Cancer Revealed by Single-Cell Sequencing. Cell 169, 1342-1356 e1316.

Field-specific reporting

Please select the one below that is the best fit for your research. If you are not sure, read the appropriate sections before making your selection.

☒ Life sciences ☐ Behavioural & social sciences ☐ Ecological, evolutionary & environmental sciences

For a reference copy of the document with all sections, see nature.com/documents/nr-reporting-summary-flat.pdf

Life sciences study design

All studies must disclose on these points even when the disclosure is negative.

Sample size	We are considering that the number of mice which we used in our experiment should be minimized because of animal right. We established mice experiment in each group (n=7 or 8) when we evaluated anti-tumor effects or cancer-specific survival. We performed at least two same experiments to show confidential and mentioned in figure legends. If we could get significant results, we have never used additional mice to prevent increasing number of sacrificed mice. When there were some differences in each group which were not significant, we retried the same experiment by increasing samples.
Data exclusions	We did not exclude any data.
Replication	We have replicated all mice experiments, which is indicated in the figure legends.
Randomization	In the tumor study of mice with therapies, we randomized all tumor-bearing mice before starting any treatment. For In vitro studies, we randomized and used a same number of samples.
Blinding	We did not perform our experiments in a blind setting because of experimental schedule and short of personnel.

Reporting for specific materials, systems and methods

We require information from authors about some types of materials, experimental systems and methods used in many studies. Here, indicate whether each material, system or method listed is relevant to your study. If you are not sure if a list item applies to your research, read the appropriate section before selecting a response.

Materials & experimental systems

n/a	Involved in the study	
☐	☒	Antibodies
☐	☒	Eukaryotic cell lines
☒	☐	Palaeontology and archaeology
☐	☒	Animals and other organisms
☒	☐	Human research participants
☒	☐	Clinical data
☒	☐	Dual use research of concern

Methods

n/a	Involved in the study	
☒	☐	ChIP-seq
☐	☒	Flow cytometry
☒	☐	MRI-based neuroimaging

Antibodies

Antibodies used	Anti-mouse CD8β mAb (clone; 53-5.8, Cat#: BE0223, Lot#: 733219O1), anti-mouse CD4 mAb (clone; GK1.5, Cat#: BE0003-1, Lot#: 689518A1), anti-mouse CTLA-4 mAb (clone; 9D9, Cat#: BE0164, Lot#: 636317J2) and anti-mouse TIGIT mAb (clone; 1G9, Cat#: BE0274) were purchased from BioXcell (West Lebanon, NH). Hybridoma for mouse PD-L1 (B7-H1) neutralizing mAb (clone 10B5) was obtained from Dr. Lieping Chen's laboratory at Yale University (Hirano et al., 2005). We diluted the antibodies by PBS when we injected to mice.<br>For western blot, we used polyclonal anti-human GPR171 (ABGENT, Lot: RB61670), Phosphorylated PLCγ-1 (Cell signaling, clone: Tyr783, 2821S), PLCγ-1 (Cell signaling, clone: D9H10, 8713S), phosphorylated p44/42 (ERK1/2) (Cell signaling, Thr202/Tyr204, clone: D13.14.4E4370S),mp44/42 MAPK (ERK1/2)(Cell signaling, clone: 137F5, 4965S), phosphorylated AKT (cell signaling, Ser473, clone: D9E, 4060S), AKT (Cell signaling, clone: C67E7, 4691S), phosphorylated ZAP70 (Tyr319/Tyr352) (Cell signaling, clone: 65E4, 2717S), ZAP70 (Cell signaling, clone: 2705S), phosphorylated CD3ζ (Tyr 142) (Cell signaling, clone: Y142, 67748S), β-actin (Cell signaling, clone: 8H10D10), CD3ζ antibody (Santa Cruz, clone: 6B10.2, sc-1239).<br>Anti-mouse CD16/32 (Biolegend, clone: 93, Cat: 1013330, Lot: B297670) was used for blocking.
Validation	Validation information of each antibody from BioXcell is follows:<br>Anti-mouse CD8β mAb (https://d2a7cdyquyl45u.cloudfront.net/tds-sheets/BE0223-tds.pdf), anti-mouse CD4 mAb (https://d2a7cdyquyl45u.cloudfront.net/tds-sheets/BE0003-1-tds.pdf), anti-mouse CTLA-4 mAb (https://d2a7cdyquyl45u.cloudfront.net/tds-sheets/BE0164-tds.pdf), anti-mouse TIGIT mAb (https://d2a7cdyquyl45u.cloudfront.net/tds-sheets/BE0274-tds.pdf).<br>Mouse PD-L1 mAb has already reported previously: Hirano, F., Kaneko, K., Tamura, H., Dong, H., Wang, S., Ichikawa, M., Rietz, C., Flies, D.B., Lau, J.S., Zhu, G., et al. (2005). Blockade of B7-H1 and PD-1 by monoclonal antibodies potentiates cancer therapeutic immunity. Cancer Res 65, 1089-1096.

Eukaryotic cell lines

Policy information about cell lines

Cell line source(s)	MC38 (colon adenocarcinoma) from Kerafast (https://www.kerafast.com/productgroup/665/mc-38-cell-line), B16F10 (mouse melanoma, https://www.atcc.org/products/crl-6475) and B16F10 expressing OVA antigen (B16-OVA) (melanoma) and Jurkat cells (human T cell lymphoblast, ATCC, https://www.atcc.org/products/crl-2899). All these cell lines were kindly provided from Dr. Lieping Chen (Yale University).<br>CT26 (colon adenocarcinoma, ATCC, https://www.atcc.org/products/crl-2638) was provided from Dr. Jill Slansky (University of Colorado).
Authentication	We performed authentication through certificate of analysis download using each lot number.
Mycoplasma contamination	All these cell lines in our laboratory were tested for Mycoplasma infection by DAPI staining upon purchase or obtaining.
Commonly misidentified lines (See ICLAC register)	We didn't use any misidentified lines.

Animals and other organisms

Policy information about studies involving animals; ARRIVE guidelines recommended for reporting animal research

Laboratory animals	Wild type C57BL/6 (H-2Kb+), BALB/c (H-2kd+) and B6D2F1 (H-2kb and d+) mice were purchased from the Jackson Laboratory (Bar Harbor, ME). OT-1 transgenic mice were kindly provided by Dr. Ross M Kedl (Department of Immunology and Microbiology, University of Colorado AMC) and were maintained in the animal facility.<br>All mice were horsed in our animal facility and the setting of mouse horsing is below: light from 6am to 8pm, keep from 72 to 75F and humidity is about 30%.<br>The Mouse Genetics Core Facility at National Jewish Health generated the GPR171lacZ/lacZ mice which are horsing in the animal facility. We used 8 to 10 weeks old female mice for our study.
Wild animals	We didn't use wild animals in our study.
Field-collected samples	We didn't use field-collected samples.
Ethics oversight	All animal care, experiments and euthanasia were performed in accordance with protocols approved by the Institutional Animal Care and Use Committee at the University of Colorado Anschutz Medical Campus. (Approved number: 132 and 461)

Note that full information on the approval of the study protocol must also be provided in the manuscript.

Flow Cytometry

Plots

Confirm that:

☒ The axis labels state the marker and fluorochrome used (e.g. CD4-FITC).

☒ The axis scales are clearly visible. Include numbers along axes only for bottom left plot of group (a 'group' is an analysis of identical markers).

☒ All plots are contour plots with outliers or pseudocolor plots.

☒ A numerical value for number of cells or percentage (with statistics) is provided.

Methodology

Sample preparation	We used mouse lymphocytes in vitro or vivo and TILs samples isolated from tumors. The protocol was mentioned in the material section.
Instrument	Samples were analyzed by a CytoFLEX (Beckman Coulter, Indianapolis, IN, USA).
Software	Data was analyzed using FlowJo software (Tree Star).
Cell population abundance	We isolated and determined immune cells by using many kinds of antibodies to keep the purity of our samples
Gating strategy	We isolated single cells (FSC-A and FSC-H) and then collect only living cells by Ghost Dye™ Red 780 (Tonbo Bioscience). To distinguish each immune cells, we used anti-mouse CD45, CD3, CD8, CD4, NK1.1, CD19, CD11b, CD11c, I-Ab, F4/80, Ly-6G, Ly-6c. To determine the cut-off line (PD-1 etc), we used iso-type control in each staining.

☒ Tick this box to confirm that a figure exemplifying the gating strategy is provided in the Supplementary Information.
